# Comment on Polak et al. Principles and Limitations of miRNA Purification and Analysis in Whole Blood Collected during Ablation Procedure from Patients with Atrial Fibrillation. *J. Clin. Med.* 2024, *13*, 1898

**DOI:** 10.3390/jcm13133671

**Published:** 2024-06-24

**Authors:** Kehinde Ross

**Affiliations:** 1 School of Pharmacy and Biomolecular Sciences, Liverpool John Moores University, Liverpool L3 3AF, UK; o.k.ross@ljmu.ac.uk; 2 Liverpool Centre for Cardiovascular Science, University of Liverpool, Liverpool John Moores University and Liverpool Heart & Chest Hospital, Liverpool L3 3AF, UK

In the current issue of the journal, the paper by Polak et al. [1] focuses on evaluating and optimising methods for the extraction of microRNAs (miRNA) from blood samples taken from atrial fibrillation (AF) patients. In the context of AF, circulating miRNAs have been profiled in both paroxysmal and persistent AF, from early work linking reduced plasma miR-150-5p levels to some aspects of AF pathogenesis [2] to a recent study that uncovered miR-20b-5p and miR-330-3p as putative cardiac-specific biomarkers that may predict AF recurrence one year after catheter ablation [3]. What makes the work of Polak and colleagues stand out is the use of left atrium blood taken during pulmonary vein isolation for both paroxysmal and persistent AF. The power of this approach lies in its potentially enhanced ability to predict the trajectory of AF compared to peripheral blood sampling. However, the authors have not compared the miRNA profile of the plasma derived from left atrium blood with the circulating miRNA profile from peripheral blood. Such comparisons would certainly be a useful addition in future studies.

Much of the study by Polak et al. focused on optimising conditions for miRNA extraction from plasma. It is worth noting that the stated elution volumes of the commercially available kits that they used vary: 14 µL for miRNeasy Serum/Plasma kit, 20 µL for miReasy Serum/Plasma Advanced kit and 2 × 40 µL for the PAXgene RNA Blood kit, all from Qiagen, Hilden, Germany. Although the authors indicate that the miRNA extraction procedures were carried out according to the manufacturer’s instructions, it would have been useful to confirm elution volumes used in each case and report the total RNA yield from each preparation. Such total amounts of RNA recovered can be crucial in planning next-generation RNA sequencing (RNAseq) experiments, for instance, which would provide comprehensive insight into the miRNA profiles of the plasma samples. Assuming the RNA was eluted in 80 µL according to the manufacturer’s procedures, the PAXgene kit gave better overall yield compared to the miReasy Serum/Plasma Advanced kit (3.4 µg total RNA from 200 µL of plasma for PAXgene versus the best of 1.4 µg RNA obtained from 150 µL plasma with the miRNeasy Serum/Plasma Advanced kit). This is important because, as Polak and colleagues demonstrated with the miRNeasy Serum/Plasma Advanced kit, starting the RNA extraction with a large volume of plasma (400 µL rather than 200 µL or 150 µL) does not guarantee a better yield. In fact, the yield was lower when 400 µL was used with the miRNeasy Serum/Plasma Advanced kit compared to using 150 µL, which the authors attributed to the clogging of the spin columns used in the procedure. That said, the PAXgene kit is designed for RNA extraction from blood rather than plasma and it would be worthwhile to determine how well the PAXgene kit performs with up to 700 µL of plasma (this being the amount of lysate supernatant recommended for PAXgene RNA spin columns).

The assessment of 84 miRNAs associated with cardiovascular disease using a PCR array from Qiagen was particularly interesting, even though it was limited to one patient. Clearly, there is a need for further studies with a large cohort. Nonetheless, it is noteworthy that several miRNAs reported to be dysregulated in AF were detected, such as miR-150-5p, miR-146a-5p and miR-222-3p, although miR-21-5p was, surprisingly, absent; for reviews of circulating miRNA in AF, the reader is referred to the references [4,5]. In any case, the miRNA profile for the patient reported by Polak and colleagues was not compared to the plasma from a control patient, making it impossible to judge the biomarker potential of the miRNAs they detected in the plasma of this AF patient. Indeed, although the authors describe the miRNAs in Figure 1 of their report as being dysregulated, using a 2-fold cut-off to demarcate some miRNAs from others, these data simply indicate that some miRNAs are more highly expressed than others when compared to the small nucleolar RNA (SNORD) reference genes used in the experiment. Hence, while the study demonstrates technical validity, more work is needed to elucidate the biological relevance of the observations.

In addition to increasing the cohort size and comparing their data to control subjects without AF, it is also worth considering next-generation sequencing (NGS) approaches rather than the PCR array. This will avoid artefacts arising from differential PCR amplification efficiency among target and reference genes [6]. Amplification-free NGS methods may offer the best insights in this regard.

Finally, it is worth noting that circulating circular RNA (circRNA) have started to emerge as potential contributes to the pathogenesis of AF [7]. Perhaps we can look forward to longitudinal studies from the Polak group where circRNAs are profiled from the left atrial and peripheral blood using their optimised RNA extraction techniques in order to uncover the predictive potential of intracardiac circRNAs as well as miRNAs in relation to AF.
